# Socio-demographic and reproductive characteristics of clients that accepted contraceptives at abortion center at the Charlotte Maxeke Johannesburg Academic Hospital (CMJAH), Johannesburg, South Africa: a cross-sectional study (January-July 2021)

**DOI:** 10.11604/pamj.2023.45.39.37441

**Published:** 2023-05-17

**Authors:** Kennedy Baffour-Duah, Lusanda Shimange-Matsose, Gbenga Olorunfemi

**Affiliations:** 1 Department of Obstetrics and Gynaecology, School of Clinical Medicine, Faculty of Health Science, University of the Witwatersrand, Johannesburg, South Africa,; 2 Division of Epidemiology and Biostatistics, School of Public Health, University of the Witwatersrand, Johannesburg, South Africa

**Keywords:** Contraceptives, induced abortion, post-abortion, family planning, Johannesburg, South Africa

## Abstract

**Introduction:**

access to family planning services is an important preventive strategy against maternal mortality as it can considerably reduce unintended pregnancies and prevent sequelae of unsafe abortion. We aimed to describe the socio-demographic and reproductive characteristics of abortion seekers and investigate factors associated with uptake of contraceptives following induced abortion at Charlotte Maxeke Johannesburg Academic Hospital, Johannesburg, South Africa.

**Methods:**

this study was a cross-sectional study among women who had legal termination of unwanted pregnancy at Charlotte Maxeke Johannesburg Academic Hospital (CMJAH), from 1^st^ January 2021 to July 2021. Questionnaires were administered to 80 consenting consecutive clients after undergoing induced abortion. Information on socio-demographic and reproductive characteristics and pattern of contraceptive uptake of the respondents were obtained. Descriptive and bivariate analysis were conducted to determine the pattern and relationship of socio-demographic and reproductive characteristics and contraceptive uptake.

**Results:**

the mean age of the 80 respondents was 25.6 ± 6.6 years. Majority of the participants were of the Black race (96.25%, n= 77/80), single (90.00%, n= 72/80), of the Christian faith (80.00%, n= 64/80) and unemployed. The median number of children alive among the clients was 1(0-2), with about 37.5% of the participants being nulliparous. About 16.25% of the participants had had at least one previous termination of pregnancy. The prevalence of post-abortion contraceptive uptake was 97.5% (95%CI: 90.36% - 99.39%, N=78/80). More than half chose injectable contraceptive (53.85%, 95%CI: 42.60% - 64.71%, N= 42/78), followed by oral contraceptive pills (21.79%, 95%CI: 13.90% - 32.49% N= 17/78). We found no association between socio-demographic and reproductive characteristics, and contraceptive uptake among the abortion clients (p values >0.05).

**Conclusion:**

the immediate post-abortion contraceptive uptake in our facility is very high. Majority of the clients accepted injectable contraceptives. The demographic and reproductive characteristics of our clients did not affect uptake of post-abortion contraception. More education is needed to improve uptake of other long-acting contraceptives that may not require frequent contact with the health facility.

## Introduction

Access to family planning services can considerably reduce unintended pregnancies and prevent sequelae of unsafe abortion [1-3]. It is therefore an important primary strategy for preventing maternal morbidity and mortality [4]. Despite the increased availability and awareness of contraceptive services, induce abortion remains a huge global public health challenge owing to poor contraceptive utilization leading to unintended pregnancies [2]. To reduce unintended pregnancies, there ought to be a conscious effort aimed at increasing the availability and accessibility of modern contraceptive use to reduce the incidence of abortion-related maternal death [1]. The provision of immediate post-abortion contraception following an induced abortion can reduce the prevalence of recurrent unwanted pregnancies, repeat abortions, and their sequelae [2].

There have been suggestions that socio-demographic characteristics such as age, ethnicity/racial differences, marital status, socio-economic status, educational status, and religious belief can play a key role in the pattern of uptake of contraception among women [5-8]. It was found that black women were less likely to use contraceptives as compared to whites in the United States of America [6]. Furthermore, greater acceptance of post-abortion contraceptives occurred among women who received post-abortion contraceptive counseling and services at health centers or maternity homes as compared to hospitals [7,9,10]. However, while some studies reported that young women are more receptive to post-abortion contraceptives, older women were more interested in post-abortion family planning in other settings [6-8]. Partner´s support and participation during the abortion process may influence the women´s acceptance and use of post-abortion contraception [11,12]. Nonetheless, the determinants of post-abortion contraceptive use vary due to contextual differences, study design and outcome of interest [6,7,13].

South Africa is a multi-ethnic country and thus may have a contraceptive uptake pattern that is influenced by ethnicity. Recognizing the significant contribution of unsafe abortion to morbidity and mortality among women of reproductive age, the South African Government liberalized the procurement of safe abortion and has over the years provided accessible, cheap, and safe abortion services to women [14]. Act 92 of the 1996 constitution affords every woman in South Africa the right to decide whether to have an early safe and legal termination of pregnancy in accordance with the provisions in the law [14-16]. Additionally, efforts have been made to make family planning services accessible to South African women to reduce unwanted pregnancies. Thus the 1996 constitution of the Republic of South Africa, Act 108 of 1996, promotes reproductive rights and the rights of access to reproductive health care [17,18]. We therefore, aimed to describe the socio-demographic and reproductive characteristics of abortion seekers and investigate factors that predict women´s use of contraception following induced abortion at Charlotte Maxeke Johannesburg Academic Hospital, Johannesburg, South Africa.

## Methods

**Study design:** this study was a cross-sectional study among women who had legal termination of unwanted pregnancy under the South African laws at Charlotte Maxeke Johannesburg Academic Hospital (CMJAH), Ward 195 from 1^st^ January 2021 to July 2021.

**Definition of terms:** induced abortion refers to deliberate intervention to terminate the pregnancy. The early first trimester here refers to gestational age less than 8 weeks. The late first trimester refers to gestational age ≥ 8 weeks to 12 weeks. Dual contraception/double Dutch method refers to the use of a barrier contraceptive along with another contraceptive method to help reduce transmission of sexually transmitted infections and prevent pregnancy respectively.

**Study site:** the study was conducted at a dedicated induced abortion clinic (ward 195) in the Department of Obstetrics and Gynecology of CMJAH Johannesburg. Ward 195 is a walk-in facility for clients who want to have a termination of pregnancy in accordance with South African laws on termination of pregnancy [14]. Charlotte Maxeke Johannesburg Academic Hospital is a Quaternary Hospital located in Johannesburg. Johannesburg is the capital city of Gauteng Province with a population of 15.5 million in 2020. Johannesburg city is mainly an urban city with a population of 5.6 million people as of 2020. The facility is manned by trained nurses and midwives who offer counseling and termination of pregnancy to the patients. There is always a supervising doctor on call who handles complicated cases. The facility has a counseling room, two functional procedure rooms and about 15 beds for the patients. There are 7 staff (4 nurses, 2 doctors, 1 counselor) and they work from 8 am to 5 pm from Monday to Friday except on public holidays. All clients who report for termination of pregnancy are first seen and assessed by the doctor and have an ultrasound scan done to confirm and date the pregnancy. The patients are then referred to see the counselors, who together with the medical team try to understand the reasons for the termination and offer options if any for the clients. Manual vacuum aspiration (MVA) with a Karman syringe is utilized for most cases of induced abortion. Some of the cases of incomplete miscarriage are also managed with MVA or misoprostol. Following the procedure, the patients are monitored at the recovery ward where they stay for 10-15 minutes for monitoring and observation and then given their preferred contraceptive. The facility performs an average of 5 abortions every day. The clients are also counseled for contraception and offered their preferred option following the abortion procedure. When the patients do not get their preferred contraceptive, they are either referred to their local clinic or asked to report another day for it. But such patients are advised to use a barrier method until they are seen at their local clinics for the preferred choice.

**Study procedure:** we recruited consecutive consenting clients from January 1^st^, 2021, to July 31^st^, 2021. We calculated our sample size based on Fischer´s formula for the survey (prevalence). A power of 80% and an error margin of 5% were assumed. We obtained the prevalence of contraceptive uptake among the abortion seekers in our center after a review of the records at the facility as 91.78%. We analyzed all the cases that were seen in 2019 and calculated the prevalence from that. We also utilized a corrected sample size for a finite population after our calculations showed a total abortion population of 240 within the period. We then added a 5% attrition rate and finally obtained a sample size of 80. Questionnaires were administered to consecutive clients who accepted to participate in the study after obtaining informed consent. The questionnaires were self-administered after the induced abortion was performed. Participants that did not understand the English language were assisted by an interpreter who understood her native language. A clinical psychologist was on standby to offer support to clients who might be distressed after completing the questionnaires. The information from the questionnaires was extracted onto a spreadsheet for analysis.

**Statistical analysis:** data was imported into Stata version 17 (StataCorp, USA) statistical software for analysis. Descriptive statistics were conducted, and categorical variables were presented as frequency and percentages while continuous variables were presented as mean and standard deviation or median and interquartile range. The prevalence of contraceptive uptake among the respondents was calculated. The association between categorical factors and uptake of post-abortion contraceptives was assessed using Pearson´s Chi-square while the association between continuous variables and the uptake of post-abortion contraceptives was assessed using the student´s t-test or Mann Whitney U. Univariable and multivariable logistic regression (with backward elimination technique) was conducted with the uptake of double Dutch contraceptive as the outcome. P-value < 0.05 was assumed to be statistically significant. Two-tailed test of the hypothesis was assumed.

**Ethical considerations:** ethical approval was obtained from the Research and Ethics Committee (Medical) of the University of Witwatersrand before the commencement of the study. (Ethics approval number: (M200802). Permission was also obtained from the Chief Executive Officer of CMJAH before the commencement of the study. Written informed consent was obtained from each participant before participating in the study. Participants were free to withdraw from the study at any time without any consequences to their care. Anonymous data was obtained and confidentiality of the data was ensured as names and other identifiers were not utilized.

## Results

In all, 80 respondents consented to participate in the study. The mean age of the respondents was 25.6 ± 6.6 years. About one-fifth of the participants were teenagers (21.25%, n= 17/80) or aged 20-24 years (22.50%, n= 18/80). Thus, nearly half of the participants were younger than 25 years (43.75%, n= 35/80). Furthermore, about one-third of the participants were aged 25-29 years (31.25%, n= 25/80). The majority of the participants were of the black race (96.25%, n= 77/80), single (90.00%, n= 72/80), of the Christian faith (80.00%, n= 64/80) and unemployed (72.15%, n= 57/80). About one-third of the participants had either secondary (35.0%, n= 28/80) or tertiary (37.5%, n=30/80) education. Thus, the majority of the participants had completed at least secondary education (72.5%, n= 58/80) (Table 1).

**Table 1:** socio-demographic characteristics of the participants who had induced abortion

Characteristics	Frequency	Percentage
**Age (mean ± SD) years**	25.6 ± 6.6	
<19	17	21.25
20-24	18	22.50
25-29	25	31.25
30-34	11	13.75
≥35	9	11.25
**Ethnicity**		
Black	77	96.25
White	2	2.50
Colored	1	1.25
**Marital status**		
Single	72	90.00
Married	4	5.00
Divorced	4	5.00
**Religion**		
Christianity	64	80.00
None	12	15.00
Traditional worshipper	4	5.00
**Educational status**		
Primary complete	3	3.75
Secondary incomplete	19	23.75
Secondary complete	28	35.00
Tertiary	30	37.50
**Employment status**		
Unemployed	57	72.15
Worked for a firm	21	26.58
Self employed	1	1.27

From Table 2, the median number of children alive among the clients was 1(0-2), with about 37.5% of the participants being nulliparous. About 16.25% of the participants had had at least one previous termination of pregnancy. Of the 13 participants that stated to have had previous termination of pregnancies, the majority (9/13, 69.2%) had one previous termination, while about 30.8% (n= 4/13) did not state the number of previous terminations. All the participants presented in the first trimester. The median gestational age at presentation was 8(7-10) weeks and about 58.8% of the participants presented at < 9 weeks of gestation. The common reasons for seeking the termination of pregnancy were “Not yet ready to be a mother” (n= 33, 41.25%), Financial reasons (n=29, 36.25%), and social reasons (n= 16, 20.0%). One client each (n=1, 1.25%) presented for induced abortion on account of sexual assault and medical reasons respectively. The majority of the clients were informed about the abortion services by healthcare workers (85.00%, n= 68/80) and all the participants have manual vacuum aspiration.

**Table 2:** reproductive and history of termination of pregnancy the respondents

Characteristics	Frequency	Percentage
**Number of children (Median, IQR)**	1(0-2)	
0	30	37.50
1	22	27.50
2	16	20.00
3	7	8.75
4	5	6.25
**Previous termination of pregnancy**		
Yes	13	16.25
No	67	83.75
**Number of previous terminations n=13**		
1	9	69.23
Not stated	4	30.77
**Gestational age at termination of pregnancy (median, IQR) Weeks**	8 (7-10)	
5	1	1.25
6	13	16.25
7	16	20.00
8	17	21.25
9	8	10.00
10	7	8.75
11	9	11.25
12	9	11.25
**Trimester at termination of pregnancy**		
Early first trimester (≤ 8 weeks)	47	58.75
Late first trimester (> 8 weeks)	33	41.25
**Sources of information about abortion services**		
Referred by health worker	68	85.00
Came on my own to find out	9	11.25
**Educational institution**	1	1.25
Heard on radio/TV/newspapers	2	2.50
**Reasons for seeking termination of pregnancy**		
Not ready to be a mother	33	41.25
Financial reasons	29	36.25
Social reasons	16	20.00
Medical doctor’s advice	1	1.25
Sexual assault	1	1.25
**Type of procedure for termination of pregnancy**		
Manual vacuum aspiration	80	100.00

IQR: interquartile range

From Table 3, about 70% (n= 56/24) of the respondents had used modern contraceptives before. Of the 24 respondents who had never used contraceptives before, 44% (n=10/24) had no reason for not using contraceptives before while about 28% (n= 7/24) were afraid of perceived side effects. About 22.50% (n= 18/80) reported that they were using modern contraceptives when they had the current unintended pregnancy. Of the 18 respondents who had failed contraceptive use, nearly half (n= 8/18, 44.44%) were on combined oral contraceptive pills. About 62.5% of the respondents will also prefer to use male condoms alongside other modern contraceptives while a lower proportion (n= 43/80, 53.75%) will be ready to use female condoms alongside other contraceptives. Out of the 80 clients who were interviewed after the abortion procedure, 78 of them accepted contraceptives. This showed that the prevalence of post-abortion contraceptive uptake was 97.5% (95%CI: 90.36% - 99.39%, N=78/80). However, 2 (2.5%, 95%CI: 0.61% - 9.64%) respondents did not accept post-abortion contraceptives. Of the two respondents yet to accept contraceptives, the reasons given were that one client did not get her preferred choice while the second person stated that she was yet to decide on the use of contraceptives.

**Table 3:** contraceptive history of the respondents

Variable	Frequency	Percentage
**Previous use of contraceptive before pregnancy**		
Yes	56	70.00
No	24	30.00
**Reasons for not using contraceptive before pregnancy, N= 24**		
No reason	10	44.00
Afraid of side effects	7	28.00
Religious and cultural reasons	4	16.00
Not sexually active	2	8.00
Don’t get time to seek for one	1	4.00
**Contraceptive use during current unintended pregnancy**		
Yes	18	22.50
No	62	77.50
**Types of contraceptive that failed, N= 18**		
Oral contraceptive pills	8	44.44
Depo provera	7	38.89
Copper T IUD	2	11.11
Condoms	1	5.56
**Counselled/informed about all the various post abortion contraceptive options**		
Yes	78	97.50
No	2	2.50
**Clients got their preferred contraceptive choice**		
Yes	78	97.50
No	2	2.50
**Will also prefer to use male condom alongside another contraceptive to prevent HIV**		
Yes	50	62.50
No	30	37.50
**Will also prefer to use female condom alongside another contraceptive to prevent HIV**		
Yes	43	53.75
No	37	46.25

**Figure 1:**
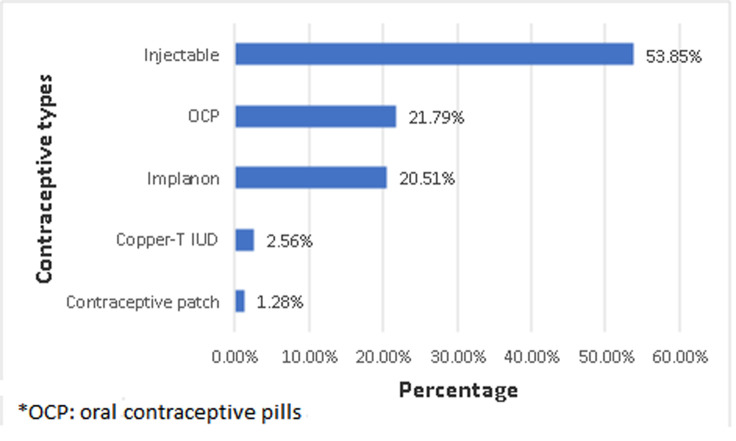
types of post-abortion contraceptives accepted by respondents

Of the 78 respondents that accepted post-abortion contraceptives, more than half chose injectable contraceptives (53.85%, 95%CI: 42.60% - 64.71%, N= 42/78), followed by oral contraceptive pill (OCPs) (21.79%, 95%CI: 13.90% - 32.49% N= 17/78), then Implanon (20.51%, 95%CI: 12.87% - 31.08%, N= 16/78). Few clients opted for copper-T intrauterine contraceptive device (IUCD) (2.56%, 95%CI: 0.63% - 9.88%, N= 2/78) and contraceptive patch (1.28%, 95% CI: 0.17% - 8.79%, N= 1/78) (Figure 1). From Table 4, there was no statistically significant association between socio-demographic characteristics and contraceptive uptake among abortion clients (P-value > 0.05). From Table 5, there was no statistically significant association between reproductive characteristics and contraceptive uptake among abortion clients (P-value > 0.05). From Table 6, there was an association between educational status and intention to utilize dual contraceptives among abortion clients (p-value = 0.028). Other socio-demographic characteristics were not statistically significant. From Table 7, there was no association between the reproductive characteristic and intention to utilize dual contraceptives among the abortion clients (p-value > 0.05).

**Table 4:** bivariate analysis of the association between contraceptive uptake and socio-demographic characteristics

Characteristics	No contraceptive uptake, N= 2	Uptake of contraceptive N= 78	Test score	P-value
**Age (mean ± SD) years**	27.5 ± 0.71	25.55 ± 6.68	t = 0.4100	0.6829
<19	0(0.00)	17 (21.79)		0.626
20-24	0(0.00)	18 (23.08)		
25-29	2 (100.00)	23 (29.49)		
30-34	0(0.00)	11(14.10)		
≥35	0(0.00)	9 (11.54)		
**Ethnicity**				
black	2 (100.00)	75 (96.15)		1.000
White	0(0.00)	2 (2.56)		
Colored	0(0.00)	1 (1.28)		
**Marital status**				
Single	2 (100.00)	70 (89.74)		0.892
Married	0 (0.00)	4 (5.13)		
Divorced	0 (0.00)	4 (5.13)		
**Religion**				
Christianity	1 (50.00)	63 (80.77)		0.362
None	1 (50.00 )	11(14.10)		
Traditional Worshipper	0 (0.00)	4 (5.13)		
**Educational status**				
Primary complete	0 (0.00)	3 (3.85)		0.734
Secondary incomplete	1 (50.00)	18 (23.08)		
Secondary complete	0 (0.00)	28 (35.90)		
Tertiary	1(50.00)	29 (37.18)		
**Employment status**				
Unemployed	1 (100.00)	56 (71.79)		1.000
Worked for a firm	0 (0.00)	21 (26.92)		
Self employed	0 (0.00)	1 (26.92)		

**Table 5:** bivariate analysis of the association between contraceptive uptake and reproductive characteristics

Characteristics	No contraceptive uptake, N= 2	Uptake of contraceptive, N= 78	Test score	P-value
**Number of children (Median, IQR)**	0.5(0-2)	1(0-2)	-0.772	0.4401
0	30	37.50		
1	22	27.50		
2	16	20.00		
3	7	8.75		
4	5	6.25		
**Primigravida**	1(50.00)	29(37.18)		0.612
≥1	1(50.00)	49 (62.82)		
**Previous termination of pregnancy**				
Yes	0 (0.00)	13 (16.67)		0.700
No	2 (100.00)	65 (83.33)		
**Gestational age at termination of pregnancy (median, IQR) weeks**	7 (6-8)	8(7-10)	-1.062	0.2883
**Trimester at termination of pregnancy**				
Early first trimester (≤ 8 weeks)	2 (100.00)	45 (57.69)		0.509
Late first trimester (> 8 weeks)	0 (0.00)	33 (42.31)		
**Sources of information about abortion services**				
Referred by health worker	2 (100.00)	66 (84.62)		1.000
Other sources	0 (0.00)	12 (15.38)		
**Reasons for seeking termination of pregnancy**				
Not ready to be a mother	0	33		0.363
Financial reasons	1	28		
Social reasons	1	15		
Medical doctor’s advice	0	1		
Sexual assault	0	1		
**Previous use of contraceptive before pregnancy**				
Yes	1	55		0.513
No	1	23		

**Table 6:** bivariate analysis of the association between double-Dutch contraceptive uptake and socio-demographic characteristics

Characteristics	Does not like dual protection with condom, N= 30 (%)	Will opt for dual protection with condom N= 50 (%)	Test score	P-value
**Age (mean ± SD) years**	25.47 ± 5.87	25.68 ± 7.06	-0.1391	0.8898
<19	5(16.67)	12(24)	2.1048	0.716
20-24	8(26.67)	10(20)		
25-29	10(33.33)	15(30)		
30-34	5(16.67)	6(12)		
≥35	2(6.68)	7(14)		
**Ethnicity**				
Black	29(96.67)	48(96)		1.000
White	1(3.33)	1(2)		
Colored	0(0)	1(2)		
**Marital status**				
Single	29(96.67)	43(86)		0.497
Married	0(0.00)	4(8)		
Divorced	1(3.33)	3(6)		
**Religion**				
Christianity	24(80)	40(80)		1.00
None	5(16.67)	7(14)		
Traditional worshipper	1(3.33)	3(6)		
**Educational status**				
Primary/incomplete secondary	6 (20.00)	25 (50.00)	7.1443	0.028
Secondary complete	9 (30.00)	10 (20.00)		
Tertiary	15 (50.00)	15 (30.00)		
**Employment status**				
Employed	6 (20.69)	16 (32.00)	1.1686	0.280
Unemployed	23 (79.31)	34 (68.00)		

**Table 7:** bivariate analysis of the association between uptake of dual contraception (male condom) and reproductive characteristics

Characteristics	Does not like dual protection with condom, N= 30	Will opt for dual protection with condom N= 50	Test score	P-value
**Number of children (median, IQR)**	1 (0-1)	1 (0-2)	-1.183	0.2369
0	12 (40.00)	18 (36.00)		0.172
1	11(36.67)	11(22.00)		
2	5(16.67)	11 (22.00)		
3	0 (0.00)	7 (14.00)		
4	2 (6.67)	3 (6.00)		
<2	23 (76.67)	29 (58.00)	2.8718	0.090
≥2	7 (23.33)	21 (42.00)		
**Previous termination of pregnancy**				
Yes	6 (20.00)	7 (14.00)	0.4960	0.481
No	24 (80.00)	43 (86.00)		
**Gestational age at termination of pregnancy (median, IQR) weeks**	7.5 (7 - 9)	8 (7 - 10)	-1.621	0.1049
**Trimester at termination of pregnancy**				
Early first trimester (≤ 8 weeks)	21 (70.00)	26 (52.00)	2.5068	0.113
Late first trimester (> 8 weeks)	9 (30.00)	24 (48.00)		
**Sources of information about abortion services**				
Referred by health worker	25 (83.33)	43 (86.00)	0.1046	0.746
Other sources	5 (16.67)	7 (14.00)		
**Reasons for seeking termination of pregnancy**				
Financial reasons	10	19	0.1780	0.915
Not ready to be a mother	13	20		
Others	7	11		
**Previous use of contraceptive before pregnancy**				
Yes	18 (60.00)	38 (76.00)	2.2857	0.131
No	12 (40.00)	12 (24.00)		

From Annex 1, the odds of utilizing the double Dutch contraceptive method among women who had completed secondary education was 5-fold as compared to the odds of intention for dual contraceptive protection among women who had tertiary education (adjusted odds ratio: 4.8, 95% CI: 1.15 - 20.09, P-value = 0.031). Furthermore, the likelihood of abortion clients with late first-trimester pregnancy (≥ 8 weeks) having the intention to utilize dual contraceptive protection with a male condom was about 4 times the likelihood among women with early first-trimester abortion (adjusted odds ratio: 3.9, 95% CI: 1.10- 13.55, P-value = 0.034). The odds of utilizing dual contraceptive generally decreases with increasing age.

## Discussion

This study was conducted to describe the socio-demographic and reproductive characteristics of abortion seekers and those clients that accepted contraceptives at the abortion center at the Charlotte Maxeke Johannesburg Academic Hospital, Johannesburg, South Africa.

**Summary of findings:** the mean age of the respondents was 25.6 ± 6.6 years, about one-fifth of the participants were teenagers (21.25%, n= 17/80) or aged 20-24 years (22.50%, n= 18/80). Nearly half of the participants were younger than 25 years (43.75%, n= 35/80). The majority of the participants were of the Black race (96.25%, n= 77/80), single (90.00%, n= 72/80), of the Christian faith (80.00%, n= 64/80) and unemployed. The median number of children alive among the clients was 1(0-2), with about 37.5% of the participants being nulliparous. About 16.25% of the participants had had at least one previous termination of pregnancy. The prevalence of post-abortion contraceptive uptake was very high: 97.5% (95%CI: 90.36% - 99.39%, N=78/80). More than half chose injectable contraceptive (53.85%, 95%CI: 42.60% - 64.71%, N= 42/78), followed by OCPs (21.79%, 95%CI: 13.90% - 32.49% N= 17/78), then Implanon (20.51%, 95%CI: 12.87% - 31.08%, N= 16/78). We found no association between socio-demographic and reproductive characteristics, and contraceptive uptake among the abortion clients.

The likelihood of utilizing the double Dutch contraceptive method among women who had completed secondary education was about 5-fold as compared to the likelihood of dual contraceptive protection among women who had tertiary education. Furthermore, abortion clients with late first-trimester pregnancy (≥ 8 weeks) had 4-fold odds of utilizing dual contraceptive protection as compared with early first-trimester abortion (adjusted odds ratio: 3.9, 95% CI: 1.10- 13.55, P-value = 0.034). The odds of utilizing dual contraceptive generally decreases with increasing age. The prevalence of post-abortion contraceptive uptake of 97.5% among our respondents was very high as compared to other studies that reported contraceptive prevalence of 73% to 77% [7,19]. The high contraceptive uptake may be due to the high-quality post-abortion family planning counseling skills of the nurses. The overall contraceptive rate in South Africa which is about 60% may also be relatively high as compared to other lower-middle-income countries (LMICs) [20]. However, nurses must be trained to avoid coercing the clients to accept contraceptives as such an attitude may trample on their reproductive rights. Of the 78 respondents that accepted post-abortion contraceptives, more than half chose injectable contraceptives (53.85%), followed by OCPs (21.79%), then Implanon (20.51%). Few clients opted for Copper-T IUCD (2.56%) and contraceptive patches (1.28%). These findings contrasted with a report of Benson *et al*. in their multicenter study where OCPs had the highest prevalence followed by condoms and then injectables [19]. Furthermore, Adelekan *et al*. also found that the oral contraceptive pill (OCP) was the commonest contraceptive followed by the injectable contraceptives among women in Gauteng province, which also contrasted with findings from our center is also located in the same province [21]. The use of reversible long-acting contraceptives such as Implanon and intrauterine contraceptive devices can be promoted especially during this Corona Virus disease (COVID-19) era to reduce contact of clients with the hospital. The aforementioned contraceptive methods have a failure rate of 0.05%, and 0.6% respectively compared to OCPs with a 0.3% failure rate with perfect use. Indeed, the majority (about 44.4%) of respondents that had failed contraceptives during this study had OCPs. Further studies can be conducted to explore reasons for the various choice of contraceptives.

While about one-fifth of our participants were teenagers, we found that almost half of the participants were younger than 25 years. This age pattern is consistent with reports from other studies that showed that women aged 20-29 years have the highest induced abortion prevalence in most countries [22]. However, Benson *et al*. found that 64% of abortion seekers in Africa were teenagers as compared to 36% in Asia [7]. Therefore, our abortion clinics must be sensitive to the needs and tendencies of young women especially with the provision of adolescent-friendly reproductive health services. Furthermore, the number of abortion seekers may be the tip of the epidemiological iceberg of unintended pregnancies in the country. More interventions should be encouraged in South Africa to promote sex education, sexual abstinence and contraceptives among the teenage population to reduce the current incidence of intended pregnancy among young women.

The majority of the participants were of the Black ethnic group (96.25%, n= 77/80). The majority of our respondents were of the Black race, possibly because people of other ethnic groups such as the White and Asians hardly patronize government facilities for their health needs, as they can afford private medical care [23]. Mosley *et al*. in their study on Abortion in Post-Apartheid South Africa reported that affluent or White women prefer to access safe abortion through private providers and non-governmental organizations (NGOs), while lower socioeconomically positioned Black women utilized the public sector, where the number of clinics and quality of services is declining [23]. It is noted that the colored population (mixed race) usually patronizes government health facilities but were very few in the present study. Further studies should be conducted to explore where and how the colored population seeks abortion care.

The majority (about 72%) of the respondents were unemployed. Thus, the inability to financially take care of the unborn child among the women may be a major reason why they seek to terminate the pregnancy although the government provides child grants. Mosley *et al*. reported that studies done in 2009 showed that black South African women of lower socioeconomic positions continue to experience considerable barriers to employment, education, and basic social services [23,24]. In a study conducted in Tigray (Ethiopia) on comprehensive abortion care, it was reported that only 36% of all the abortion clients seen were married [25]. This finding is in line with our study where 90% of the clients were single and not married. The majority of the participants/respondents who seek abortion had completed at least secondary education (72.5%, n= 58/80). Mosley *et al*. found that respondents who completed their secondary education or who had any tertiary education were less likely to report abortion as 'always wrong' [24]. This may suggest that women of higher educational status are likely to seek safe induce abortion as we found in our study. Women of low educational status may be seeking abortion elsewhere which may be unsafe.

About 16.25% of the participants have had at least one previous termination of pregnancy. Similarly, about 23% of women seeking induced abortion in a Nigerian community reported having had one or more previous abortions [26]. Failure to offer safe and effective post-abortion contraceptives has been shown to increase the risk of repeated unwanted pregnancies [27,28]. In the United Kingdom, about 39% of women who had an abortion in 2018 had one or more previous abortions [29]. This was an increase from 33% in 2008. Similarly, studies in Australia have shown that 36.6% of current abortion seekers had previous termination of pregnancies [30]. This means that our cases of repeat abortions are lower as compared to the figures elsewhere, possibly because of the high incidence of post-abortion contraceptives at the center.

All the participants presented for abortion care in the first trimester. The median gestational age at presentation was 8(7-10) weeks and about 58.8% of the participants presented at < 9 weeks of gestation. Our findings were consistent with the study by Prata *et al*. in Ethiopia where they reported that on average, women presenting for safe termination had a mean uterine size of nine weeks gestation [25]. The common reasons for seeking the termination of pregnancy were not being ready to be a mother (n= 33, 41.25%). Alemu *et al*. similarly found that the majority (51.9%) of the participants who had induced abortion in their study in Ethiopia were not ready and the index pregnancy was unintended [3]. Only a few clients presented for abortion services on account of sexual assault (n= 1/80, 1.25%) and medical reasons (n= 1, 1.25%). This may be the only legal reason for the termination of pregnancy in some countries [31]. Thus, if abortion services were restricted to only medical and or crime-related conditions more than 97% of those in need of abortion for other reasons would have been denied the service. The majority of the clients were informed about the abortion services by healthcare workers (85.00%, n= 68/80) and all the participants had manual vacuum aspiration. This means more publicity should be done so that participants will not visit quacks for abortion services.

Our study showed that there was no statistically significant association between socio-demographic and reproductive characteristics and uptake of contraceptives. This pattern may be because of the cross-sectional study design in which a very high percentage of the respondents had uptake of contraceptives. A case-control study of an equal number of cases (respondents who had contraceptives) and cases (respondents who did not take contraceptives) may suffice. Surprisingly, there was a higher likelihood of utilizing double Dutch (dual) contraceptives to prevent human immunodeficiency virus (HIV) infection among secondary school levers as compared to tertiary level holders. It is believed that clients with tertiary education should be more enlightened about HIV preventive behaviors which was not the case in this study. Only two out of every three respondents stated that they will use double Dutch contraceptive techniques. Since the HIV prevalence is high in South Africa, we suggest that more efforts aimed at promoting dual protection (contraception and HIV) should be encouraged during post-abortion contraceptive counseling.

**Limitations and strengths of the study:** to our knowledge, this is the first study to describe the socio-demographic and reproductive characteristics of the abortion seekers at our center. A limitation of the study was the cross sectional-design as a case-control study design might be better to recruit more respondents that did not accept post-abortion contraceptives. Social desirability concerns may occur among the respondents while answering the questions. However, we attempt to obviate this by giving self-administered questionnaires.

**Funding:** Gbenga Olorunfemi was funded by Glaxo Smithkline (GSK) Africa Non-Communicable Disease Open Lab through the DELTAS Africa Sub-Saharan African Consortium for Advanced Biostatistics (SSACAB) training programme. (Grant number: D1702270-01). The views expressed in this publication are those of the author(s) and not necessarily those of GSK as GSK was not part of the conception, design and dissemination of the findings of the research.

## Conclusion

The immediate post-abortion contraceptive uptake in our facility is very high. The majority of the clients accepted injectable contraceptives. It appears that the demographic and reproductive characteristics of our clients did not affect the uptake of post-abortion contraception. Further studies (possibly case-control studies or even qualitative/explorative studies are necessary). More education is needed to improve the uptake of other long-acting contraception that may not require frequent contact with the health facility.

### What is known about this topic

- *Socio-demographic characteristics such as age, ethnicity/racial differences, marital status, socio-economic status, educational status, and religious belief can play key role in the pattern of uptake of contraception among women;*
- *It was found that black women were less likely to use contraceptive as compared to whites in the United States of America;*
- *There is greater acceptance of post-abortion contraceptives among women who received post-abortion contraceptive counselling and services at health centres or maternity homes as compared to hospitals*.

### What this study adds

- *South Africa is a multi-ethnic country and thus may have been expected to have contraceptive uptake pattern that is influenced by ethnicity; however, the demographic and reproductive characteristics of our clients did not affect uptake of post-abortion contraception*;
- *The immediate post-abortion contraceptive uptake in our facility is very high*.

## Annex

**Annex 1**: multivariable regression model of some determinants of intention to use double contraceptive protection among women who seek abortion (PDF - 160Kb)
